# Curcumin attenuates collagen-induced inflammatory response through the “gut-brain axis”

**DOI:** 10.1186/s12974-017-1047-7

**Published:** 2018-01-06

**Authors:** Yannong Dou, Jinque Luo, Xin Wu, Zhifeng Wei, Bei Tong, Juntao Yu, Ting Wang, Xinyu Zhang, Yan Yang, Xusheng Yuan, Peng Zhao, Yufeng Xia, Huijuan Hu, Yue Dai

**Affiliations:** 1 Department of Pharmacology of Chinese Materia Medica, China Pharmaceutical University, 24 Tong Jia Xiang, Nanjing, 210009 China; 2 Department of Pharmacology and Physiology, Drexel University College of Medicine, 245 N. 15th Street, Philadelphia, PA 19102 USA

**Keywords:** Curcumin, Collagen-induced arthritis, Gut-brain axis, Unilateral cervical vagotomy, α7 nAChR, Nodose ganglion neurons

## Abstract

**Background:**

Previous studies have demonstrated that oral administration of curcumin exhibited an anti-arthritic effect despite its poor bioavailability. The present study aimed to explore whether the gut-brain axis is involved in the therapeutic effect of curcumin.

**Methods:**

The collagen-induced arthritis (CIA) rat model was induced by immunization with an emulsion of collagen II and complete Freund’s adjuvant. Sympathetic and parasympathetic tones were measured by electrocardiographic recordings. Unilateral cervical vagotomy (VGX) was performed before the induction of CIA. The ChAT, AChE activities, and serum cytokine levels were determined by ELISA. The expression of the high-affinity choline transporter 1 (CHT1), ChAT, and vesicular acetylcholine transporter (VAChT) were determined by real-time PCR and immunohistochemical staining. The neuronal excitability of the vagus nerve was determined by whole-cell patch clamp recording.

**Results:**

Oral administration of curcumin restored the imbalance between the sympathetic and parasympathetic tones in CIA rats and increased ChAT activity and expression of ChAT and VAChT in the gut, brain, and synovium. Additionally, VGX eliminated the effects of curcumin on arthritis and ACh biosynthesis and transport. Electrophysiological data showed that curcumin markedly increased neuronal excitability of the vagus nerve. Furthermore, selective α7 nAChR antagonists abolished the effects of curcumin on CIA.

**Conclusions:**

Our results demonstrate that curcumin attenuates CIA through the “gut-brain axis” by modulating the function of the cholinergic system. These findings provide a novel approach for mechanistic studies of anti-arthritic compounds with low oral absorption and bioavailability.

## Background

Rheumatoid arthritis (RA) is a serious autoimmune disease and affects 0.5–1.0% of adults worldwide [1]. Increasing evidence indicates that the autonomic nervous system (ANS) plays an important role in the regulation of an abnormal immune response and inhibition of inflammation [2, 3]. The ANS modulates cytokine production mainly through the cholinergic anti-inflammatory pathway [4] in which the efferent vagus nerve, the neurotransmitter ACh, and its receptors (especially α7 nicotinic ACh receptor, α7 nAChR) are indispensable [5–7].

Curcumin has been used historically as a spice and medicinal herb in India and China [8]. Considerable evidence has suggested that curcumin possesses diverse bioactivities [9–12]. In recent years, numerous studies have shown that oral administration of curcumin significantly ameliorated collagen-induced arthritis (CIA) [13, 14]. A clinical trial has shown that curcumin is a safe and effective agent for RA patients [15]. However, pharmacokinetic studies have shown that its bioavailability is very poor which raise questions about how curcumin produces an anti-inflammatory effect [16–18].

Previous studies in our laboratory have suggested that curcumin exerts such effects in a gut-dependent manner [19]. The gut, as a sensory organ, senses luminal content including ingestion of food, microorganisms, gastrointestinal secretions, and pharmaceuticals. The signals arising from the lumen are transmitted though enteric and vagal pathways to the central nervous system, which in turn sends efferent signals to peripheral tissues or organs [20]. Previous studies strongly suggest that the vagus nerve mediates the direct gut-brain-peripheral communication [21, 22]. Anti-inflammatory responses initiated by vagal nerve activation were followed by release of ACh. The released ACh subsequently led to activation of the α7 nAChR on macrophages or immune cells, which are located in the RA joint or in the spleen [21–24]. It has been reported that curcumin could improve cholinergic system dysfunction by downregulating AChE activity and increasing ChAT activity, subsequently exerting anti-inflammatory and neuroprotective effects in the brain [25–27]. Based on these previous reports, we hypothesized that increasing cholinergic tone might be a potential anti-arthritic mechanism of curcumin. The present study demonstrated that oral administration of curcumin increased heart rate variability, enhanced ACh biosynthesis and release in the gut, brain, and synovium, and increased excitability of nodose ganglion neurons. These findings indicate that curcumin produces anti-CIA effects through the “gut-brain axis” and reveal a novel approach for the treatment of patients with RA and with other immune-mediated inflammatory diseases such as inflammatory bowel disease (IBD). The present study also provides an intriguing paradigm for mechanistic studies of anti-inflammatory compounds with low oral absorption and bioavailability.

## Methods

### Animals

Female Wistar rats, 6–8 weeks, weighing 150 ± 20 g, were purchased from Shanghai Super B&K Laboratory Animal Corp. Ltd. (Shanghai, China) and Charles River (MA, USA). They were maintained in standard laboratory chow with tap water ad libitum and under climate-controlled environment. All in vivo studies were carried out in compliance with China Pharmaceutical University Animal Care and Use Committee guidelines. All in vitro studies were carried out in compliance with the Drexel University College of Medicine Animal Care and Use Committee guidelines.

### Collagen II (CII)-induced arthritis (CIA) in rats and curcumin treatment

Arthritis was induced in rats by immunization with an emulsion of chicken type II collagen (CII, Sigma-Aldrich Co., St. Louis, MO, USA) and complete Freund’s adjuvant (CFA, Becton Drive Co., NJ, USA) as previously reported [28]. Briefly, rats were intradermally injected with 200 μl of this emulsion at the base of the tail on day 0. Seven days after the primary immunization, rats were boosted with an emulsion of CII and incomplete Freund’s adjuvant (IFA, Becton Drive). On day 14, rats were randomly assigned to normal group, model group, and curcumin group (100 mg/kg, Nanjing Zelang Medical Technology Co., Nanjing, China). Curcumin was suspended in 0.5% sodium carboxymethyl cellulose (CMC-Na) and orally administered daily 2 weeks. Normal and model group rats were orally administered vehicle according to the same schedule.

### Unilateral cervical vagotomy (VGX) and induction of CIA in rats

A VGX operation was performed 4 days before the induction of CIA [29]. In brief, rats were anesthetized and a ventral cervical midline incision was made to expose the left cervical vagus trunk, which was next ligated with 4–0 silk sutures and divided. The skin was closed with three sutures. The left nerve trunk of sham rats was exposed and isolated from surrounding tissue without being transected. After 4 days, CIA was induced as described above and the day of immunization was marked as day 0. Seven days later, rats were boosted with the second injection. On day 14, sham rats were divided into the following groups: sham group, model group, curcumin (100 mg/kg) group, leflunomide (2 mg/kg, Suzhou Changzheng-Xinkai Pharmaceutical Co., Suzhou, China) group, and nicotine (300 μg/kg, Nanjing Zelang Medical Technology) group. VGX rats were divided into VGX group, V-model group, and V-curcumin (100 mg/kg) group. Curcumin and leflunomide were orally administered, and nicotine was intraperitoneally injected for consecutive 2 weeks. Other groups were given an equal volume of vehicle with the same schedule and route of administration.

### Effects of pretreatment with nicotinic ACh receptor (nAChR) antagonists on curcumin-treated CIA rats

CIA was induced as describe above. Two weeks after the first immunization, rats were randomly assigned to the following groups: (1) normal group, model group, curcumin (100 mg/kg) group, mecamylamine (1 mg/kg, Sigma) group, curcumin (100 mg/kg) + mecamylamine (1 mg/kg) group, hexamethonium (4 mg/kg, Sigma) group, curcumin (100 mg/kg) + hexamethonium (4 mg/kg) group, and leflunomide (2 mg/kg) group; (2) normal group, model group, curcumin (100 mg/kg) group, α-bungarotoxin (1 μg/kg, Abcam Inc., Cambridge, UK) group, curcumin (100 mg/kg) + α-bungarotoxin (1 μg/kg) group, and nicotine (300 μg/kg) group. Curcumin and leflunomide were suspended in 0.5% CMC-Na and orally administered daily 2 weeks. Antagonists were dissolved in 0.9% NaCl solution and intraperitoneally injected 10 min before curcumin administration. Nicotine was dissolved in 0.9% NaCl solution and intraperitoneally injected. Normal and model group rats were orally administered 0.5% CMC-Na in the same schedule.

### Assessment of CIA

CIA was assessed by body weight, paw swelling, and arthritis index (AI) scores. A plethysmometer was used to measure the volumes of paws. The clinical scores were evaluated blindly as follows: 0 point: no arthritis; 1 point: swelling in one type of joint; 2 points: swelling in two types of joint; 3 points: swelling in three types of joint; and 4 points: swelling of the entire paw. The AI scores was the sum of four paws, with the maximum score of 16 for each rat. The ankles of rats were photographed immediately after rats were sacrificed with ether anesthesia. After continuous treatment for 2 weeks, the hind paws were photographed to evaluate the morphological changes in the ankle of rats. On the same day, the representative radiographs of the hind paws of the CIA rats were obtained to examine the bone erosion and joint destruction.

### Histological analysis

The right ankles were fixed in 10% buffered formalin for 48 h. The joints were then decalcified in 10% EDTA, embedded in paraffin, cut into 5-μm serial sections, and stained with hematoxylin and eosin (H&E). The histopathological changes in the joints were examined using an optical microscope and graded on a scale of 0–3 (0 = none changes, 1 = mild changes, 2 = moderate changes, and 3 = severe changes) by a pathologist blinded to the experimental groups [28].

### Heart rate (HR), blood pressure, and heart rate variability (HRV) analysis

Heart rate (HR), blood pressure, and HRV summary variables in time and frequency domain were assessed on day 27. Blood pressure was first measured using BP-6 Animal Non-Invasive Blood Pressure Measuring System (Chengdu Tme Technology Co, Ltd., Chengdu, China). Systolic (SP) and diastolic pressures (DP) were recorded, and mean arterial pressure (MP) was calculated according to the following formula: MP = (SP + 2 × DP)/3. Rats were then anesthetized by intraperitoneal injection of 10% urethane and placed in the supine position on a temperature-controlled cushion and allowed to breathe spontaneously. The mean HR and HRV parameters were obtained from electrocardiogram (ECG) by BL420S biological signal acquisition system (Chengdu Tme Technology Co, Ltd.). The needle electrodes were subcutaneously inserted in rat left forelimb and both hind limbs, respectively. ECG was measured for 10 min before and 1 h after treatment to obtain the HRV parameters. The time domain includes standard deviation of all R-R intervals (SDNN) and square root of the mean of the sum of the squares of differences between adjacent R-R intervals (RMSSD). The frequency domain data collected from each period of 10 min underwent spectral analysis by using a fast Fourier transform algorithm to determine the high-frequency power (HF) and low-frequency power (LF) components. The range for HF was 0.15–0.4 Hz and the range for LF was 0.04–0.15 Hz.

### Assessment of ChAT and AChE activities

The synovium tissues were weighed, and an adequate amount of 0.9% NaCl solution was added to obtain 10% (*w*/*v*) homogenates. Blood samples were collected from rats under ether anesthesia using orbital eye bleeds. Then, after clotting for 20 min at room temperature, blood was centrifuged for 20 min at a speed of 3000 rpm at 4 °C, and the serum was collected. The enzymatic activities of ChAT and AChE were measured by kits according to the manufacturer’s instructions (Bioswap Biotech Co., Wuhan, China). Activities were expressed as units per gram weight of synovium tissue and units per liter of serum, respectively.

### Quantitative PCR (qPCR)

Total RNAs from the small intestine, brain, and synovium were isolated using TRIzol reagent (Invitrogen, CA, USA) according to the manufacturer’s instructions. RNA was reversely transcribed to cDNA using HiScript RT SuperMix (Vazyme Biotech Co., Nanjing, China) and then analyzed for the expressions of high-affinity choline transporter 1 (CHT1), ChAT, and VAChT by Ace qPCR SYBR Green Master Mix (Vazyme Biotech) using MyiQ2 Detection System (Bio-Rad Laboratories, Hercules, CA) [19]. The following primers (Sangon Biotech Co., Shanghai, China) were used: for CHT1, 5′-CTACATTCCCCTACGTGGTCC-3′ (sense) and 5′-AGGCCGATGGCATAAGAGAAG-3′ (antisense); for ChAT, 5′-CCAGTTCTTTGTCTTGGATGTT-3′ (sense) and 5′-GGACGCCATTTTGACTATCTTT-3′ (antisense); for VAChT, 5′-GTGCCCATTGTTCCCGACTA-3′ (sense) and 5′-CTTTCTGTGGGGTAGCGAGG-3′ (antisense); and for β-actin, 5′-CCCATCTATGAGGGTTACGC-3′ (sense) and 5′-TTTAATGTCACGCACGATTTC-3′ (antisense). All values were expressed relative to the expression of the reference gene, β-actin, and the relative expression of each gene was determined according to the 2^-ΔΔCt^ method.

### Immunohistochemistry

Small intestines, brains, and synovium tissues were harvested, fixed in 10% formalin, and then embedded in paraffin and sliced into 5-μm-thick sections (Reichert HistoSTAT, USA). The sections were incubated with the first primary antibodies against ChAT and VAChT overnight at 4 °C, and examined with DAB Envision System (Dako, Glostrup, Denmark) according to the manufacturer’s instructions.

### Measurement of cytokines in serum

The concentrations of TNF-α, IL-1β, IL-6, IL-17A, IFN-γ, IL-4, IL-10, and TGF-β in serum were detected using enzyme-linked immunosorbent assay (ELISA) kits (Dakewe Biotech Co., Shenzhen, China) according to the manufacturer’s instructions. The absorbance was measured at 450 nm.

### Cell culture

Primary nodose ganglion neuronal cultures were prepared from adult female rats as described previously with some modifications [30]. Briefly, rats were deeply anesthetized with overdose of isoflurane. The nodose ganglia of the vagus nerve were carefully removed and washed in Hank’s balanced saline solution (HBSS) (Invitrogen) (in mM: 137 NaCl, 5.4 KCl, 0.4 KH_2_PO4, 1 CaCl_2_, 0.5 MgCl_2_, 0.4 MgSO_4_, 4.2 NaHCO_3_, 0.3 Na_2_HPO_4_, and 5.6 glucose). The ganglia were then incubated on an incubator shaker (New Brunswick Scientific, NJ, USA) for 30 min at a speed of 135 rpm at 37 °C in HBSS containing papain (15 U/ml, Worthington Biochemical, NJ, USA) and collagenase from clostridium histolyticum (5 × 10^−4^ g/ml, Sigma), rinsed three times with HBSS, and placed in culture medium containing Neurobasal A (Invitrogen), 2% B-27 (Life Technologies, NY, USA), 2% heat-inactivated horse serum, 2% fetal calf serum, 0.2 mM L-glutamax, 100 U/ml penicillin, and 100 μg/ml streptomycin (Invitrogen). The fragments were mechanically dissociated by gently triturating with a pipette. The dispersed cells were seeded onto 12-mm poly-D-lysine and laminin (Sigma)-coated coverslips at a density of 3000 cells per well. Neurons were cultured at 37 °C in a humidified atmosphere containing 5% CO_2_.

### Electrophysiological recordings

Standard whole-cell recordings were performed at room temperature using an EPC 10 amplifier and PatchMaster software (HEKA Elektronik, Lambrecht, Germany) as described previously [31]. Electrode resistances ranged between 3 and 6 MΩ with series resistances of 6–15 MΩ and were compensated to the maximal current amplitude. For current-clamp recordings, the membrane voltage was held at − 65 mV; the bath solution was Tyrode’s solution containing (in mM) 140 NaCl, 5 KCl, 2 CaCl_2_, 1MgCl_2_, 10 HEPES, and 5.6 glucose, pH adjusted to 7.36 with NaOH (osmolality ~ 292 mmol/kg). The intracellular solution contained (in mM) 140 KMeSO_4_, 2MgCl_2_, 1 EGTA, 10HEPES, 3 Na_2_ATP, and 0.3 Na_2_GTP, pH adjusted to 7.4 with KOH (osmolality ~ 292 mmol/kg). Action potentials were recorded in the current-clamp mode at a membrane potential around − 65 mV. A current injection was used to evoke 2 to 3 action potentials and remained constant throughout the recording. Only one neuron was recorded from each coverslip.

### Electrophysiological data analysis

Off-line data analysis was processed using PatchMaster (HEKA) and Origin 8.1 software (OriginLab, Northampton, USA).

### Statistical analysis

Data were presented as mean ± S.E.M.. Statistical significance was assessed by one-way analysis of variance (ANOVA) followed by post hoc Tukey’s test. An 0.05 (*p* < 0.05) was considered statistically significant. The data and statistical analysis comply with the recommendations on experimental design and analysis in pharmacology.

## Results

### Curcumin attenuated CIA in rats

**Fig. 1:**
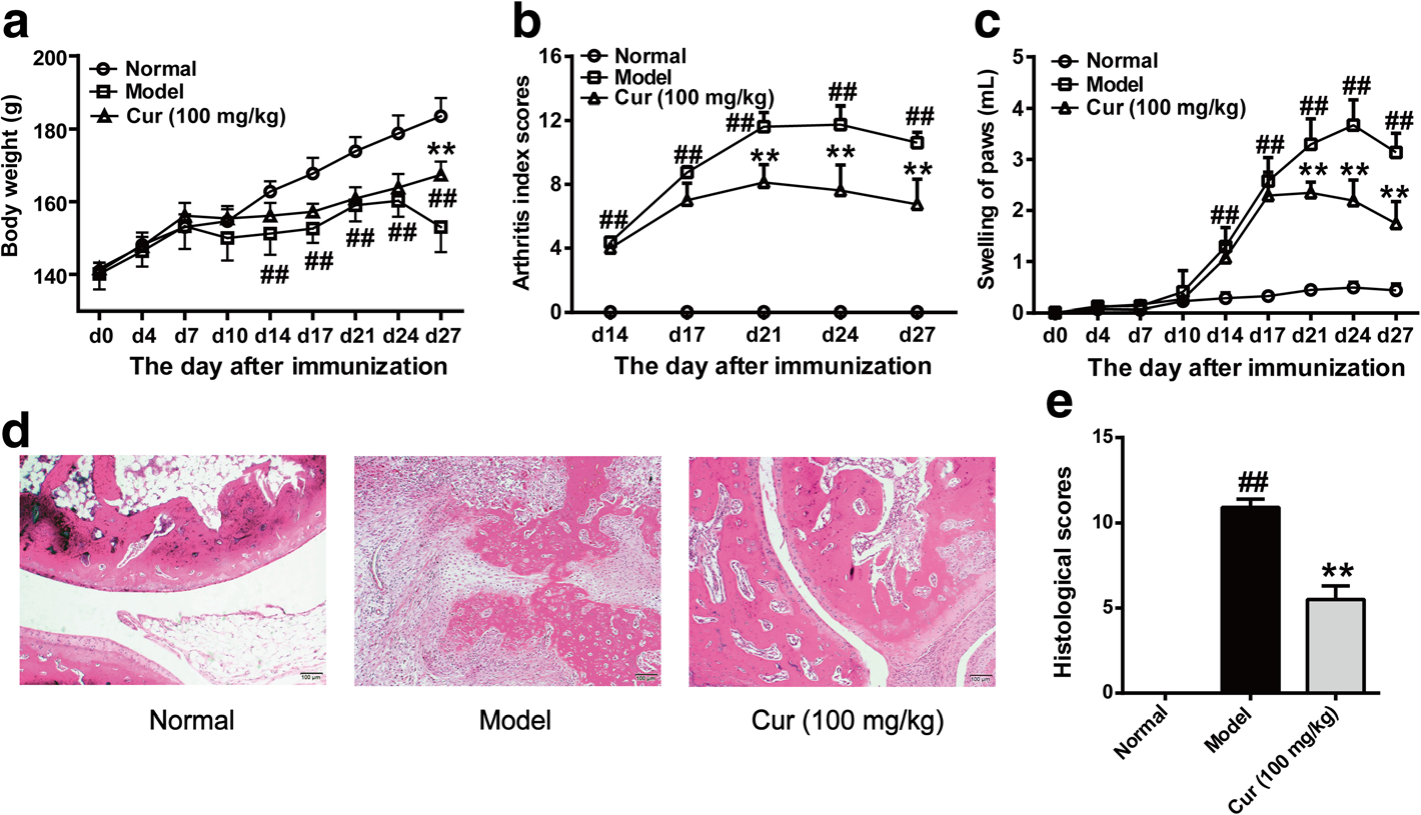
Effect of curcumin on collagen-induced arthritis (CIA) in rats. Rats were intradermally injected with type II collagen (CII) to induce CIA. Curcumin (Cur, 100 mg/kg) was orally administered daily for 14 consecutive days. **a** Body weight changes. **b** Arthritis index scores. **c** Hind paws swelling. The volumes of hind paws were all measured using a plethysmometer on indicated days. **d** Histologic examinations of the ankle joint sections. The scores of inflammatory cell infiltration, synovial hyperplasia and congestion, pannus formation, and cartilage and bone erosion. **e** The total histological scores were summarized. Data were shown as means ± S.E.M. for each group (*n* = 6). ^##^*p* < 0.01 vs. normal group; **p < 0.01 vs. model group

We and others have shown that curcumin produces an anti-arthritic effect in a mouse model of CIA and in a rat model of adjuvant-induced arthritis [13, 19]. To confirm the effect of curcumin in the CIA model in rats, we generated a rat model of CIA. Following the development of CIA, the body weight, arthritis index (AI) scores, and hind paw swelling were measured to evaluate the severity of arthritis. After treatment for 2 weeks, rat ankles in each group were removed to evaluate pathomorphological changes. We observed that the CIA rats developed arthritis, showing body weight loss, erythema, swelling of all fours, joint stiffness, and deformed paws and ankles (Fig. 1a–c). Histological analysis demonstrated marked inflammatory cell infiltration, synovial hyperplasia, and cartilage and bone erosion in ankle joints (Fig. 1d, e). Curcumin 100 mg/kg (an effective dose used in our previous study) [19] drastically attenuated CIA, as illustrated by the notable amelioration of the paw swelling, AI scores, and histological changes (Fig. 1). These results confirmed our previous findings that curcumin has anti-arthritic effects.

### Curcumin increases the cholinergic function in CIA rats

**Fig. 2:**
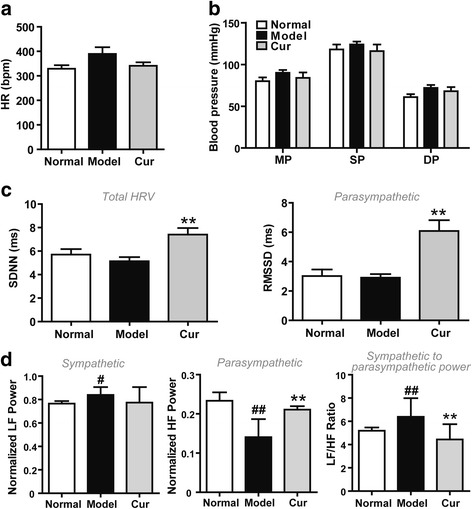
Effect of curcumin on the cholinergic system function in collagen-induced arthritis (CIA) rats. Rats were intradermally injected with type II collagen (CII) to induce CIA. Curcumin (Cur, 100 mg/kg) was orally administered daily 2 weeks, and the heart rate (HR), blood pressure, and heart rate variability (HRV) were assessed 1 h after treatment on day 27. **a** Effect of Cur on HR in CIA rats. **b** Effect of Cur on blood pressure in CIA rats. **c** Effect of Cur on HRV summary variables in time domain in CIA rats. **d** Effect of Cur on HRV summary variables in frequency domain in CIA rats. SDNN standard deviation of all R-R intervals, RMSSD square root of the mean of the sum of the squares of differences between adjacent R-R intervals, LF low-frequency power, HF high-frequency power. Data were shown as means ± S.E.M. for each group (*n* = 6). ^#^*p* < 0.05, ^##^*p* < 0.01 vs. normal group; **p < 0.01 vs. model group

A recent clinical study demonstrated that vagus nerve stimulation attenuates cytokine production and rheumatoid arthritis (RA), suggesting a therapeutic potential for vagus nerve stimulation in RA [32, 33]. To explore whether the autonomic nervous system (ANS) is involved in the anti-arthritic effect of curcumin, electrocardiographic recordings were performed. We measured cardiovascular reflex (heart rate (HR), blood pressure) that is associated with sympathetic nervous activity, and heart rate variability (HRV), which is related to vagus nerve activity [34]. CIA rats showed a reduced parasympathetic and increased sympathetic tone, but no changes in HR and blood pressure (Fig. 2a, b), which was in line with the previous clinical report [2]. Interestingly, curcumin had no significant influence on HR and blood pressure (Fig. 2a, b). However, it markedly increased HRV of CIA rats, restored the imbalance between sympathetic and parasympathetic tones by enhancing SDNN, RMSSD, and normalized high-frequency power (HF) (Fig. 2c, d). These results suggest an increase in vagus nerve activity. Since vagus nerve function is directly correlated with the activity of the cholinergic anti-inflammatory pathway, these data suggest that curcumin ameliorated the cholinergic system function in CIA rats.

### Curcumin enhances ACh biosynthesis and transport in CIA rats

**Fig. 3:**
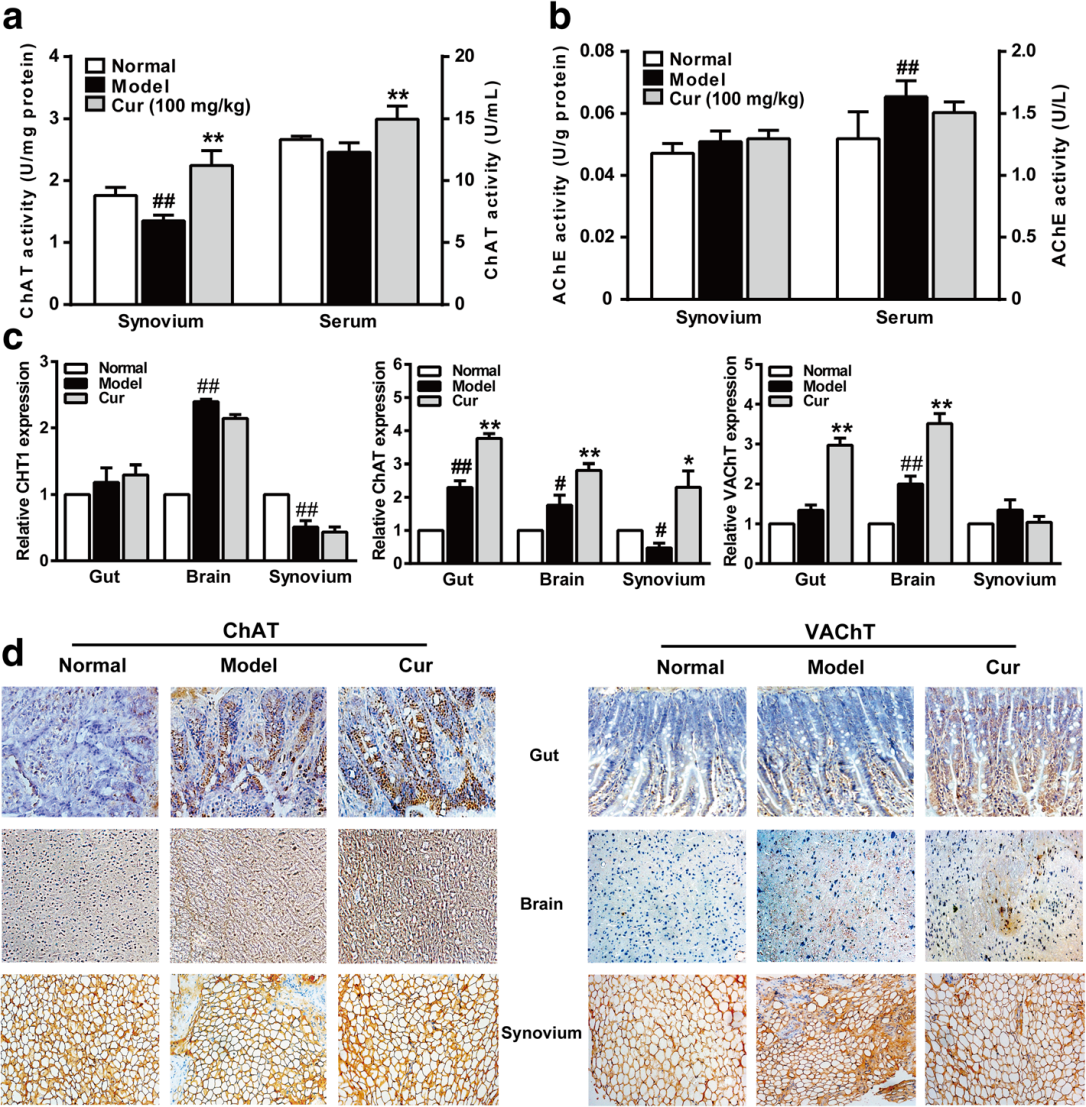
Effect of curcumin on acetylcholine biosynthesis and transport in collagen-induced arthritis (CIA) rats. Rats were intradermally injected with type II collagen (CII) to induce CIA. Curcumin (Cur, 100 mg/kg) was orally administered daily 2 weeks, and serum, gut, brain, and synovium of rats were collected 1 h after treatment on day 28. The enzymatic activities of choline acetyltransferase (ChAT) and acetylcholinesterase (AChE) in synovium and serum were measured by ELISA kits. The relative expression of high-affinity choline transporter 1 (CHT1), ChAT, and vesicular acetylcholine transporter (VAChT) in gut, brain, and synovium were analyzed by quantitative real-time PCR. The β-actin was used as the internal control. **a** The enzymatic activity of ChAT. **b** The enzymatic activity of AChE. **c** The relative expressions of CHT1, ChAT, and VAChT. **d** The expressions of ChAT and VAChT were assessed by immunohistochemical staining from paraffin-embedded tissue sections. Data were shown as means ± S.E.M. for each group (*n* = 6). ^#^*p* < 0.05, ^*##*^*p* < 0.01 vs. normal group; *p < 0.05, **p < 0.01 vs. model group

Synthetic enzyme, ChAT, and hydrolytic enzyme, AChE, have been used as markers of cholinergic system activity. Activation of parasympathetic nerves leads to the release of the cholinergic transmitter ACh, which is rapidly hydrolyzed into choline and acetic acid after release from nerve endings. The choline is taken up by the high-affinity choline transporter 1 (CHT1) into the pre-synaptic nerve where it combines with acetyl-CoA through the catalytic action of ChAT to subsequently synthesize ACh. The newly synthetized ACh is loaded by the vesicular acetylcholine transporter (VAChT) into secretory synaptic vesicles for ACh release [4, 35]. We first measured the activities of ChAT and AChE in the knee joint synovium tissues and serum and examined the mRNA and protein expression of CHT1, ChAT, and VAChT in the gut, brain, and synovium. Curcumin markedly enhanced ChAT activity and increased expression of ChAT and VAChT (Fig. 3a, c, and d). However, curcumin had no effect on AChE activity and CHT1 expression (Fig. 3b–d), suggesting that curcumin promoted the biosynthesis and transport of ACh without affecting the hydrolyzation of ACh and uptake of choline in the gut and brain. However, the oral bioavailability of curcumin is poor. Previous studies in our laboratory have shown that even when curcumin (100 mg/kg) was orally administrated continuously to arthritis rats for 14 days, the *C*_max_ was still very low (about 0.03 μM), but curcumin shows obvious accumulation in the gut (about 70 μM) [19]. These results indicate a possible pathway that the gut might be the initial site of action.

### Vagus nerve integrity is required for curcumin-induced anti-CIA effect

**Fig. 4:**
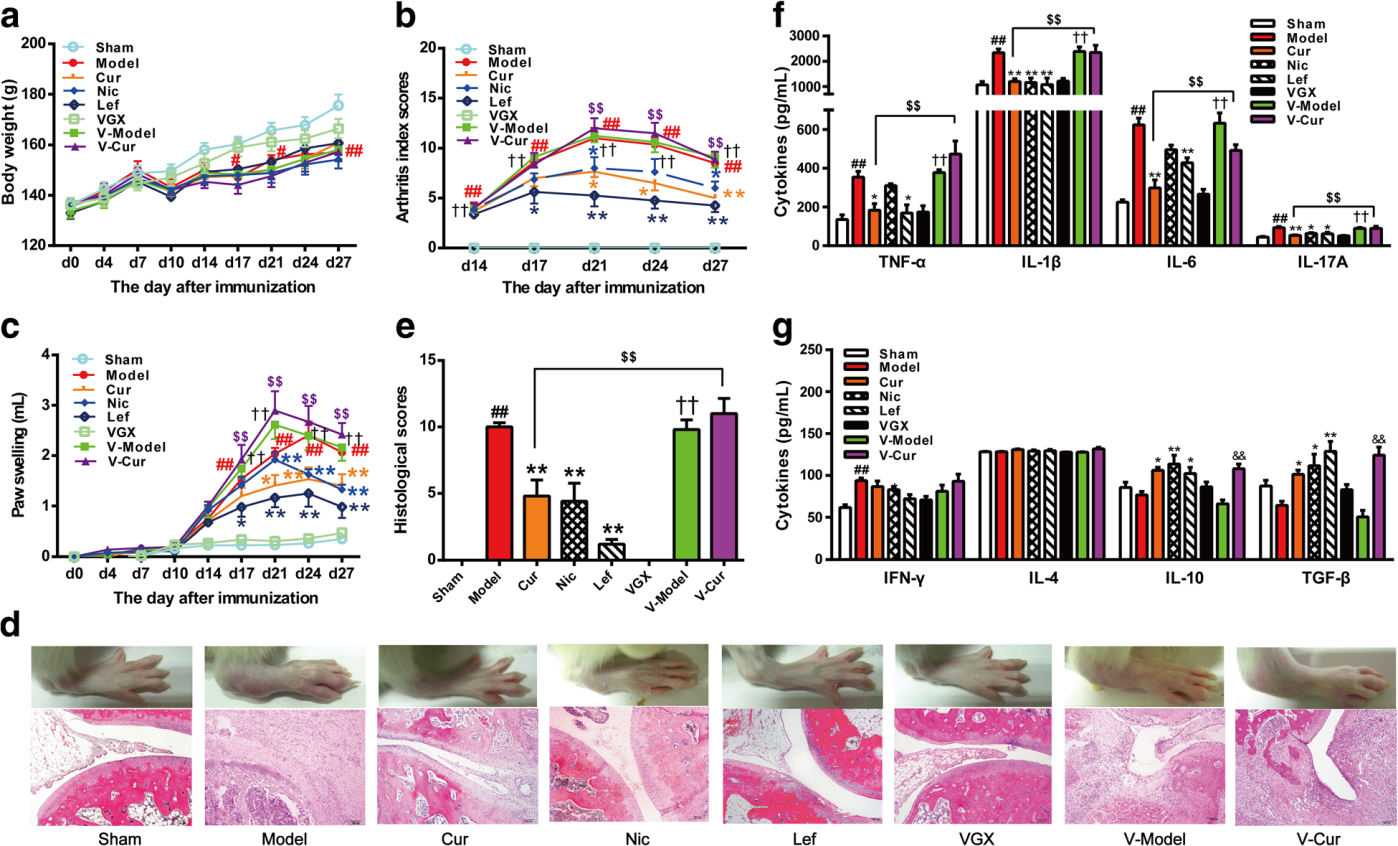
Unilateral cervical vagotomy blocked the inhibitory effect of curcumin on collagen II-induced arthritis in rats. Rats were subjected to unilateral cervical vagotomy (VGX) 4 days before collagen II-induced arthritis (CIA) was induced. Fourteen days after first immunization, curcumin (Cur, 100 mg/kg) and leflunomide (Lef, 2 mg/kg) were administered orally, and nicotine (Nic, 300 μg/kg) was intraperitoneally injected for 2 weeks. Rats were anesthetized by inhalation of ether 1 h after treatment on day 28, and serum was obtained. The cytokine levels were determined by ELISA. **a** Body weight changes. **b** Arthritis index scores. **c** Hind paws swelling. The volumes of hind paws were measured using a plethysmometer. **d** Representative ankle morphologies and pathomorphological changes of the ankle sections (H&E staining, original magnification ×100) in each group. After treatment for consecutive 2 weeks, the ankles of CIA rats were photographed. And then ankles of rats were removed, fixed, decalcified, and then stained with H&E to evaluate histological changes. **e** The total histological scores of ankle joint sections were summarized. **f** Serum concentrations of pro-inflammatory cytokines (TNF-α, IL-1β, and IL-6) and Th17 cell-related cytokine (IL-17A). **g** Serum concentrations of Th1, Th2, and Treg-related cytokines (IFN-γ, IL-4, IL-10, and TGF-β). Data were shown as means ± S.E.M. for each group (*n* = 6). *p < 0.05, **p < 0.01 vs. model group; ^#^*p* < 0.05, ^##^*p* < 0.01 vs. sham group; ^††^*p* < 0.01 vs. VGX group; ^&&^*p* < 0.01 vs. V-model group; ^$$^*p* < 0.01 vs. Cur group

It has been reported that the vagus nerve mediates the anti-inflammatory action of pharmacological agents [36, 37]. To investigate the involvement of the cholinergic system in the anti-arthritic effects of curcumin, rats were subjected to unilateral cervical vagotomy (VGX) before CIA was induced. The arthritic symptoms in V-Model (VGX-CIA) rats were slightly exacerbated, as illustrated by an increase of 7.5% in paw swelling compared with Model (sham-CIA) rats. Curcumin (100 mg/kg) produced a protective effect on ongoing CIA (Fig. 4a–e). However, in V-model (VGX-CIA) rats, curcumin failed to suppress the paw swelling and histological scores (Fig. 4a–e). These data demonstrate that the anti-arthritic effect of curcumin (100 mg/kg) was markedly diminished after VGX, indicating that the integrity of the peripheral cholinergic nerve is essential for the curcumin-induced protective effects observed in CIA rats.

Pro-inflammatory or T cell-related cytokines participate in the pathological process of RA. Increased cytokine levels might exacerbate systemic inflammatory responses and aggravate arthritis symptoms. To further investigate the effects of VGX on the anti-arthritic actions of curcumin, we measured serum levels of inflammatory cytokines using ELISA kits. Curcumin (100 mg/kg) decreased TNF-α, IL-1β, IL-6, and IL-17A and increased IL-10 and TGF-β, suggesting that curcumin effectively attenuated inflammatory responses and modulated abnormal immune responses (Fig. 4f). Interestingly, VGX completely reversed the inhibitory effect of curcumin on levels of TNF-α, IL-1β, IL-6, and IL-17A, but had no significant effect on IFN-γ, IL-4, IL-10, and TGF-β levels (Fig. 4g).

### Vagus nerve integrity is required for curcumin-induced upregulation of ChAT and VAChT

**Fig. 5:**
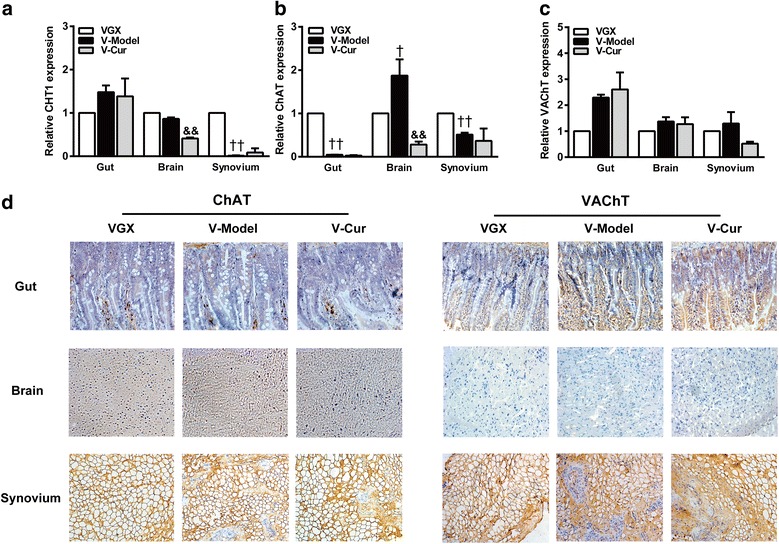
Unilateral cervical vagotomy blunted the enhancement of curcumin on acetylcholine biosynthesis and transport. Rats were subjected to unilateral vervical vagotomy (VGX) 4 days before collagen-induced arthritis (CIA) was induced. Fourteen days after first immunization, curcumin (Cur, 100 mg/kg) were administered orally for 2 weeks. Gut, brain, and synovium were collected 1 h after treatment on day 28. The relative expression of high-affinity choline transporter 1 (CHT1), choline acetyltransferase (ChAT), and vesicular acetylcholine transporter (VAChT) were analyzed by quantitative real-time PCR. The β-actin was used as the internal control. **a** The relative expression of CHT1. **b** The relative expression of ChAT. **c** The relative expression of VAChT. **d** The expressions of ChAT and VAChT were assessed by immunohistochemical staining from paraffin-embedded tissue sections. Data were shown as means ± S.E.M. for each group (*n* = 6). ^†^*p* < 0.05, ^††^*p* < 0.01 vs. VGX group; ^&&^*p* < 0.01 vs. V-model group

To further determine whether VGX alters the effects of curcumin on ACh biosynthesis and transport in CIA rats, qPCR and immunohistochemical staining were performed in the gut, brain, and synovium. Data showed that the effects of curcumin on ChAT and VAChT expression were markedly blunted by vagotomy (Fig. 5a–d). These results further confirm the crucial role of vagus nerve integrity and suggest the existence of a “gut-brain axis.”

### Curcumin increases neuronal excitability in nodose ganglion neurons from vagus nerve

**Fig. 6:**
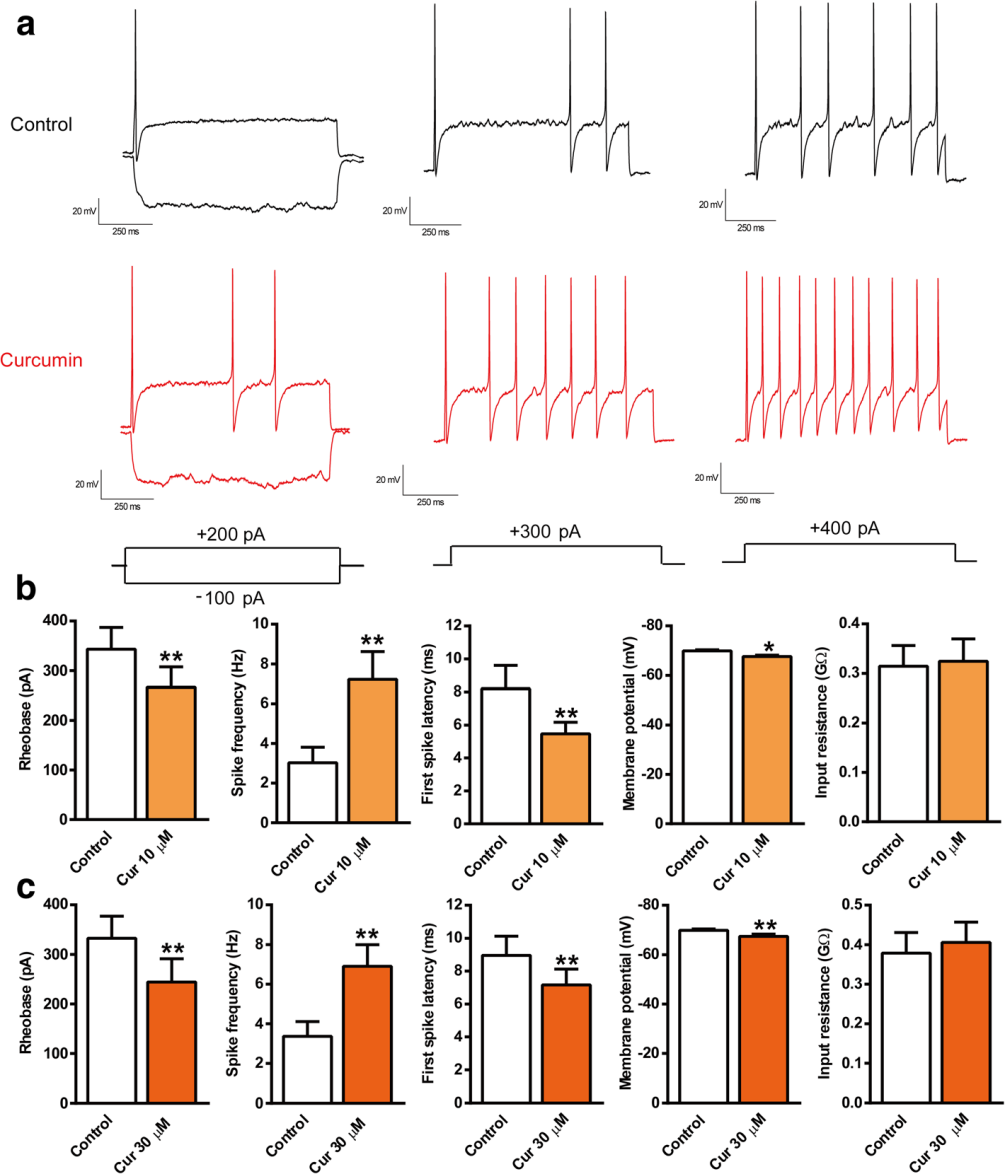
Effect of curcumin on neuronal excitability in nodose ganglion neurons. The vagal nodose ganglion neurons were obtained from adult rats, and electrophysiological recording was performed from 12 to 48 h. The membrane potential was held at − 65 mV, and action potentials were generated by injecting current. **a** Representative action potentials recorded before (control) and 5 min after treatment of curcumin (Cur, 10 and 30 μM). **b**, **c** Summary of Cur (10 and 30 μM) induced changes in rheobase, spike frequency, first spike latency, membrane potential, and input resistance in nodose ganglion neurons. Data were shown as means ± S.E.M. for each group (*n* = 12–15). *p < 0.05, **p < 0.01 vs. control group

To determine whether curcumin directly stimulates the vagus nerve, we collected nodose ganglia of the vagus nerve from adult rats. Primary nodose ganglion neurons were cultured for 12 to 48 h for action potential recordings. Action potentials were generated by current injection, and the firing rate was increased when current injection increased. Curcumin (10 and 30 μM) after 5-min perfusion decreased rheobase (threshold current to firing), increased spike frequency (spike numbers in a depolarizing pulse, 1 s), decreased first spike latency (the time between initiating current injection to the occurrence of the first action potential), and increased membrane potential (depolarized the neurons) without changes on the steady-state input resistance, suggesting curcumin increases neuronal excitability (Fig. 6). These data indicate that curcumin directly activates the vagus nerve and promotes the release of ACh, which functions as an anti-inflammatory parasympathetic neurotransmitter through the cholinergic anti-inflammatory pathway.

### Curcumin-induced anti-CIA efficacy is mediated by nAChR

**Fig. 7:**
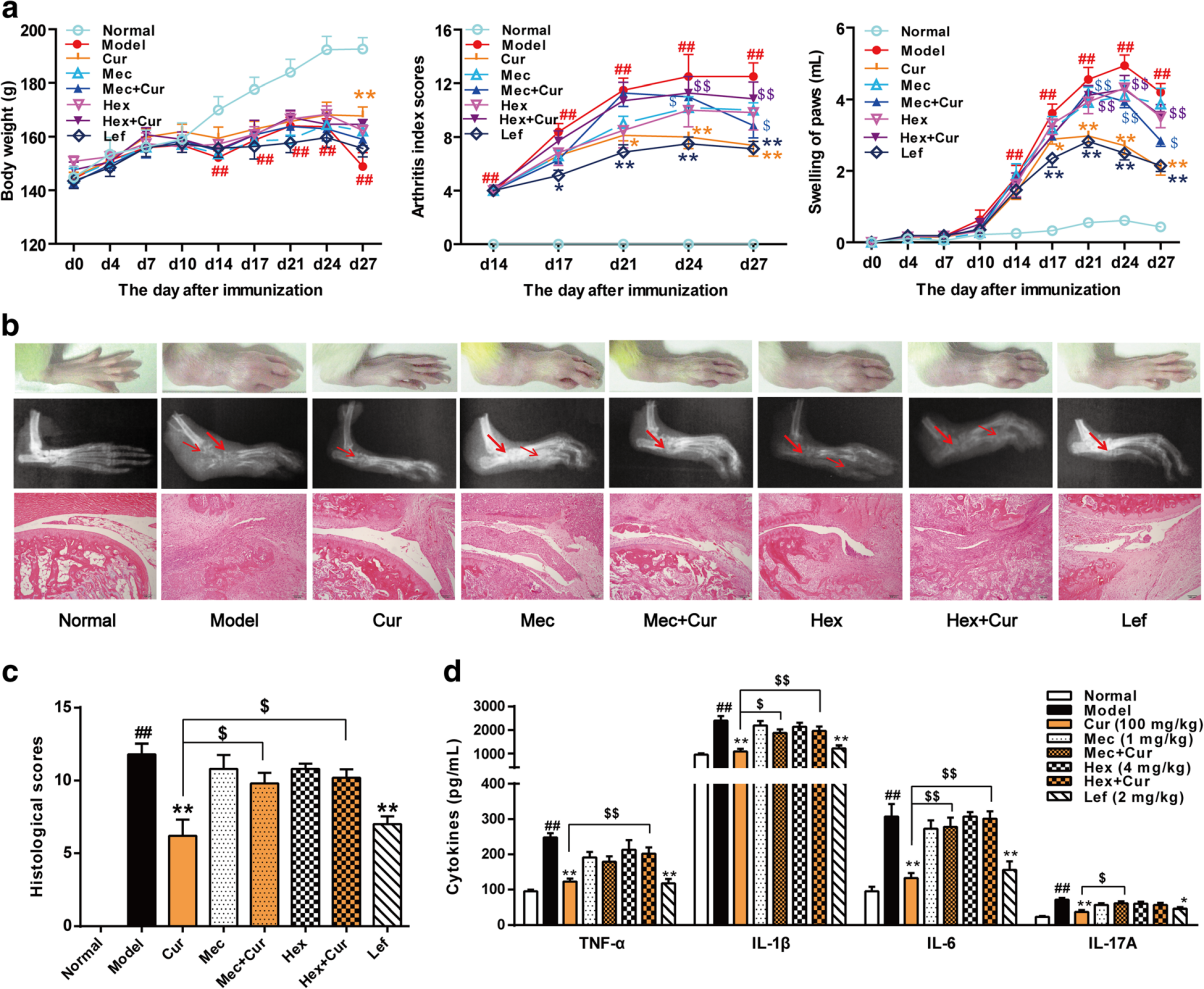
Effect of curcumin on collagen-induced arthritis (CIA) is mediated by the cholinergic receptors. Rats were intradermally injected with type II collagen (CII) to induce CIA. Curcumin (Cur, 100 mg/kg) and leflunomide (Lef, 2 mg/kg) were orally given daily 2 weeks. Mecamylamine hydrochloride (Mec, 1 mg/kg) and hexamethonium chloride (Hex, 4 mg/kg) were intraperitoneally injected 10 min before Cur administration. Blood was collected 1 h after treatment on day 28, and the cytokines in serum were assessed by ELISA. **a** Body weight changes, arthritis index scores, and hind paw swelling. **b** Representative hematoxylin and eosin (H&E)-stained sections of ankles (original magnification ×100), representative morphology photographs and radiographs of hind paws of CIA rats. Arrows indicate areas of bone destruction. **c** Histological scores of ankle joint sections. **d** The levels of cytokines (TNF-α, IL-1β, IL-6, and IL-17A) in serum were assessed by ELISA. Data were shown as means ± S.E.M. for each group (*n* = 6). ^##^*p* < 0.01 vs. normal group; *p < 0.05, **p < 0.01 vs. model group; ^$^*p* < 0.05, ^$$^*p* < 0.01 vs. Cur group

To determine whether the effect of curcumin on CIA is mediated by nAChR, different cholinergic receptor antagonists (mecamylamine, a central and peripheral neuronal nAChR antagonist; hexamethonium, a peripheral neuronal nAChR antagonist, respectively) were used [38]. Curcumin (100 mg/kg) markedly ameliorated the clinical symptoms and histological changes of CIA. Leflunomide (2 mg/kg), a commercially available immunosuppressant disease-modifying anti-rheumatic drug (DMARD) used for RA treatment, showed similar effects to those observed in the curcumin-treated group. Neither of the nAChR antagonists when administered alone had significant effects on CIA. Nevertheless, when used in combination with curcumin, hexamethonium almost completely reversed the anti-arthritic effects of curcumin while mecamylamine partially inhibited the effects (Fig. 7a–c). To investigate whether nAChR antagonists attenuate the curcumin-induced decrease in cytokine production in CIA rats, we assessed cytokine levels of TNF-α, IL-1β, IL-6, and IL-17A in serum from CIA rats. Curcumin reduced the serum concentration of inflammatory cytokines in CIA rats. However, both cholinergic receptor antagonists almost completely reversed curcumin-induced inhibition of production of TNF-α, IL-1β, and IL-6 (Fig. 7d).

### Curcumin-induced anti-CIA effect is mediated by α7 nAChR

**Fig. 8:**
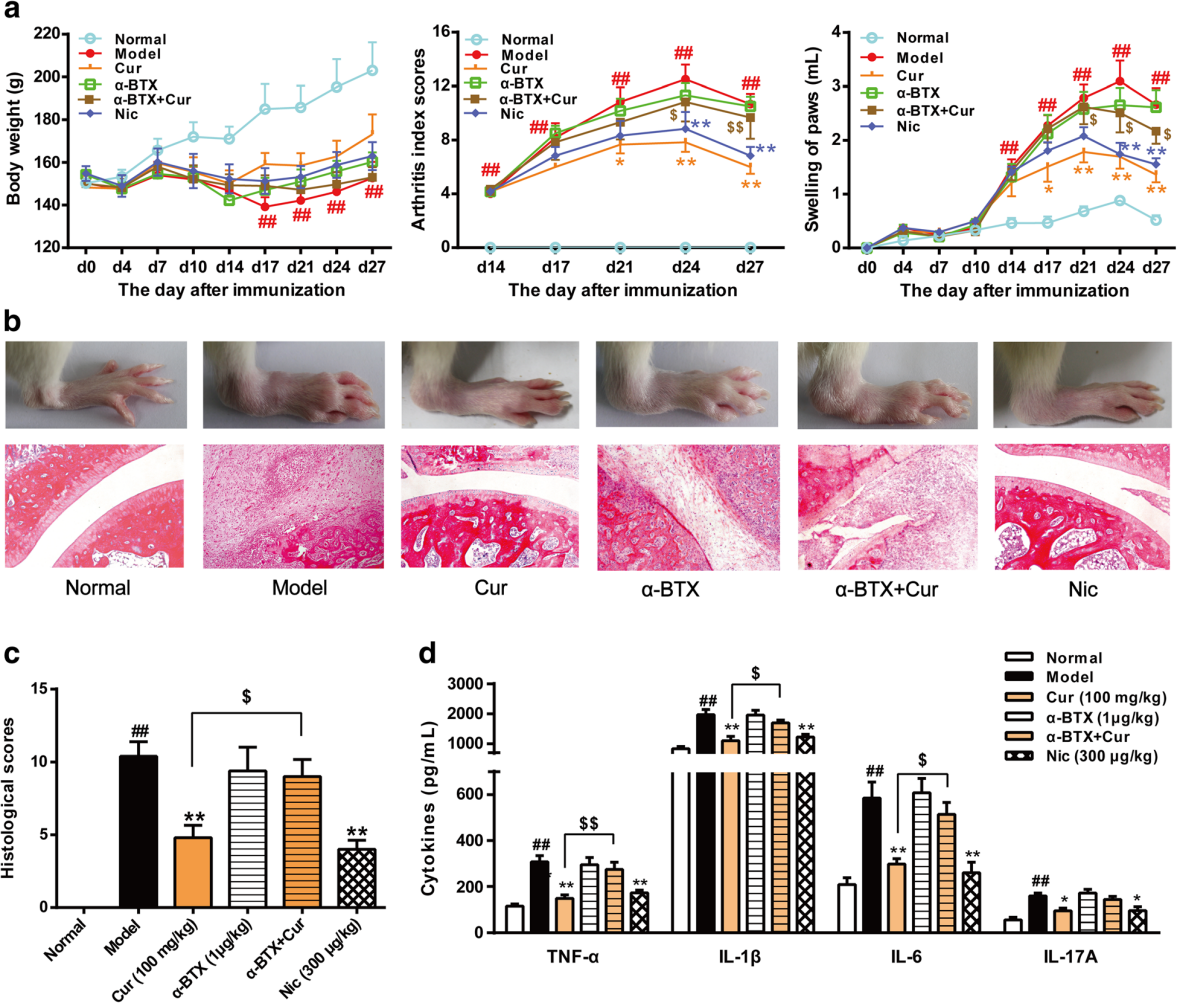
Effect of curcumin on collagen-induced arthritis (CIA) is mediated by α7 nAChR. Rats were intradermally injected with type II collagen (CII) to induce CIA. Curcumin (Cur, 100 mg/kg) were orally given daily 2 weeks. Selective α7 nAChR antagonist α-bungarotoxin (α-BTX, 1 μg/kg) was intraperitoneally injected 10 min before Cur administration. Blood was collected 1 h after treatment on day 28, and the cytokines in serum were assessed by ELISA. **a** Body weight changes, arthritis index scores and hind paw swelling. The volumes of hind paws were measured using a plethysmometer. **b** Representative hematoxylin and eosin (H&E)-stained sections of ankles (original magnification ×100), representative morphology photographs of hind paws of CIA rats. **c** Histological scores of ankle joint sections. **d** The levels of cytokines (TNF-α, IL-1β, IL-6 and IL-17A) in serum were assessed by ELISA. Data were shown as means ± S.E.M. for each group (*n* = 6). ^##^*p* < 0.01 vs. normal group; *p < 0.05, **p < 0.01 vs. model group; ^$^*p* < 0.05, ^$$^*p* < 0.01 vs. Cur group

Previous studies have suggested that α7 nAChR is an important component of the cholinergic anti-inflammatory pathway [3, 5, 29]. To further determine whether α7 nAChR mediates the anti-CIA effects of curcumin, we tested the effect of α-bungarotoxin (α-BTX), a selective α7 nAChR antagonist, on the anti-arthritic effect of curcumin. α-BTX, (1 μg/kg), which had no effect on CIA, abolished anti-CIA action of curcumin (100 mg/kg) (Fig. 8).

### Curcumin-induced modulation of neuronal excitability is mediated by α7 nAChR in nodose ganglion neurons

**Fig. 9:**
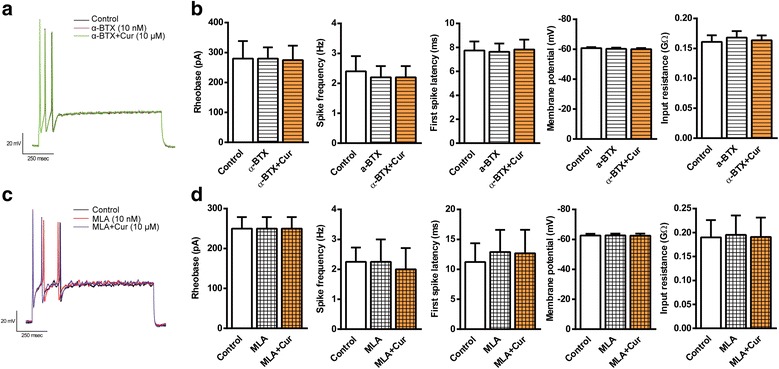
Effect of curcumin on neuronal excitability is mediated by α7 nAChR in nodose ganglion neurons. The vagal nodose ganglion neurons were obtained from adult rats, and electrophysiological recording was performed from 12 to 48 h. The membrane potential was held at − 65 mV, and action potentials were generated by injecting current. Curcumin (Cur, 10 μM) induced modulation of neuronal excitability before and 5 min after bath application of two selective α7 nAChR antagonists α-bungarotoxin (α-BTX, 10 nM) and methyllycaconitine citrate (MLA, 10 nM), respectively, were recorded. **a**, **c** Representative action potentials. **b**, **d** Summary of changes in rheobase, spike frequency, first spike latency, membrane potential, and input resistance in nodose ganglion neurons. Data were shown as means ± S.E.M. for each group (*n* = 5–9)

To determine whether curcumin-induced increase in neuronal excitability is also mediated by α7 nAChR, we first examined effects of α7 nAChR antagonists (α-BTX and methyllycaconitine citrate (MLA)) on action potentials. Neither of the antagonists used alone had significant effects on action potentials (Fig. 9). However, curcumin-induced modulation of action potentials was completely blocked by α-BTX (10 nM) and MLA (10 nM), respectively. These results demonstrated that α7 nAChR plays a crucial role in the activation of the vagus nerve by curcumin.

## Discussion

Rheumatoid arthritis (RA) is a systemic refractory arthropathy that impacts patient quality of life. Up to now, the fundamental pathophysiology of RA has not been fully elucidated [39, 40]. However, evidence is mounting that the nervous system plays a vital role in the occurrence and development of RA [3]. The neural-endocrine-immune system constitutes a complex network and participates in maintaining homeostasis in organisms. It is well known that the nervous system is the regulating center of inflammatory and immune responses [41]. Growing evidence indicates that the immune and nervous system maintain extensive interactions. The bidirectional communication between them could regulate the inflammatory responses in diseases such as RA.

The autonomic nervous system (ANS) is divided into the sympathetic nervous system (SNS) and the parasympathetic nervous system (PNS), which act either in synergy or in opposition to control life functions like body temperature, heart rate, blood pressure, and gastrointestinal motility to maintain homeostasis [42]. A clinical trial has demonstrated that the ANS is imbalanced in RA patients, with an increased sympathetic and reduced parasympathetic tone [43]. Moreover, recent reports have suggested that the PNS plays an important role in chronic immune-mediated inflammatory diseases. Activation of the PNS by either pharmacological or electrical stimulation of the vagus nerve attenuates inflammatory diseases [44–46]. Pharmacological cholinoceptor antagonists or vagotomy attenuate these cholinoceptor agonist-induced anti-inflammatory effects [47–50]. Hence, it is possible to target the neural pathways for treatments of excessive inflammation and autoimmune conditions.

Clinical studies have shown that curcumin can bring relief to osteoarthritis and RA [13–15]. However, the pharmacokinetic studies have indicated that curcumin has poor bioavailability due to poor absorption, rapid metabolism, and rapid systemic elimination [16–18]. This contradiction between the therapeutic efficacy and poor pharmacokinetics of curcumin has yet to be resolved and required further investigation.

We have previously demonstrated that oral administration of curcumin attenuates adjuvant-induced arthritis through a gut-dependent mechanism [19]. In the present study, we confirmed the anti-arthritic effects of curcumin and found that curcumin increases the activity of ChAT and regulates the cholinergic system function by reducing sympathetic tone and increasing parasympathetic tone. There is a great body of evidence indicating that the vagus nerve is a vital component of the inflammatory reflex. Once it is damaged, the cholinergic anti-inflammatory pathway cannot function normally [2, 3, 28, 51, 52].

Our data showed that unilateral cervical vagotomy completely abolished the anti-arthritic effects of curcumin, suggesting a critical role of an intact vagus nerve. Since the vagus nerve contains both afferent and efferent fibers, vagotomy removes both afferents and efferents. Further investigation is needed to elucidate the role of afferent fibers in anti-arthritic effect of curcumin. The electrophysiological results demonstrated that curcumin markedly increased neuronal excitability in nodose ganglion neurons, which further indicates that curcumin may exert its effect by directly activating the vagus nerve, suggesting the anti-arthritic effect of curcumin is mediated by the “gut-brain axis.” We further examined whether ACh receptors are involved in the curcumin-induced modulation of the cholinergic system function. Our results showed that the α7 nAChR antagonist markedly blocked the effects of curcumin on collagen-induced arthritis (CIA) and action potentials, suggesting that α7 nAChR mediates curcumin-induced modulation of neuronal excitability and CIA. Interestingly, recent studies have shown that curcumin exhibits regulatory effects on the gut microbiota in menopausal model, hepatic steatosis, DSS-induced colitis [53–56], and that the gut microbiota is involved in the pathogenesis of arthritis [57–59]. Based on these findings, it is possible that curcumin affects the cholinergic anti-inflammatory pathway through the gut-brain axis via modulation of gut microbiota.

## Conclusions

In conclusion, we have demonstrated that curcumin suppressed the inflammatory response and attenuated CIA by targeting the “gut-brain axis.” Our findings therefore reveal that targeting the gut cholinergic anti-inflammatory pathway could be a promising therapeutic intervention for patients with RA and with other inflammatory disorders (such as IBD) characterized by imbalanced ANS. The present study also identified a novel mechanism of curcumin-induced anti-arthritic effects and provided an intriguing paradigm for the subsequent mechanistic studies of natural products with low oral absorption and bioavailability.

## Abbreviations

AI: Arthritis index
ANS: Autonomic nervous system
CHT1: Choline transporter 1
CIA: Collagen-induced arthritis
H&E: Hematoxylin and eosin
HRV: Heart rate variability
IBD: Inflammatory bowel disease
PNS: Parasympathetic nervous system
qPCR: Quantitative PCR
RA: Rheumatoid arthritis
SNS: Sympathetic nervous system
VGX: Unilateral cervical vagotomy
α7 nAChR: α7 nicotinic ACh receptor
